# Why measure tumours?

**DOI:** 10.1007/s00247-014-3148-0

**Published:** 2015-01-01

**Authors:** Øystein E. Olsen

**Affiliations:** 1 Radiology Department, Great Ormond Street Hospital for Children NHS Foundation Trust, London, WC1N 3JH UK; 2 Institute of Child Health, University College London, London, UK

**Keywords:** Neoplasm, Staging, Radiology, Reliability, Validity, Measurements, Lung nodules

## Abstract

This article questions the scientific justification of ingrained radiologic practices exemplified by size measurements of childhood solid tumours. This is approached by a critical review of staging systems from a selection of paediatric oncological treatment protocols. Local staging remains size-dependent for some tumour types. The consequent stage assignment can significantly influence treatment intensity. Still, the protocols tend not to give precise guidance on how to perform scans and standardise measurements. Also, they do not estimate or account for the inevitable variability in measurements. Counts and measurements of lung nodules are, within some tumour groups, used for diagnosis of metastatic disease. There is, however, no evidence that nodule size is a useful discriminator of benign and malignant lung nodules. The efficacy of imaging depends chiefly on observations being precise, accurate and valid for the desired diagnostic purpose. Because measurements without estimates of their errors are meaningless, studies of variability dependent on tumour shape and location, imaging device and observer need to be encouraged. Reproducible observations make good candidates for staging parameters if they have prognostic validity and at the same time show little covariation with (thereby adding new information to) the existing staging system. The lack of scientific rigour has made the validity of size measurement very difficult to assess. Action is needed, the most important being radiologists’ active contribution in development of oncological staging systems, attention to standardisation, knowledge about errors in measurement and protection against undue influence of such errors in the staging of the individual child.

## Introduction

Quantities give confidence, and perhaps consolation. As radiologists we wish to categorise, score and measure. Describing and reporting on an imaging-detected mass lesion without providing estimates of its size would seem to rock our very foundations. The universal criteria for assessment of tumour response in preclinical trials are based solely on lesion numbers and sizes [1]. Staging and treatment of childhood neoplasms may depend on size measurements from diagnostic images: the size of the primary and the size and number of lung nodules. But is the ruler a useful tool? Are measurements reproducible, accurate and meaningful? Or, in a few more words: if both of us measure the same tumour, will we report reasonably similar sizes? When I estimate tumour volumes, is it reasonable to assume that most of my estimates are a negligible distance away from the true volumes? Last, does tumour size represent independent additional information that improves the care for the individual child? The three questions are logically connected. It may be possible to perfectly reproduce measurements that are far from a true volume, and it is conceivable that we can have both reproducible and accurate estimates that do not help to optimally stratify the child’s clinical care. Conversely, evidence that tumour volume really is useful for guiding therapy is negligible if we cannot produce reliable and accurate estimates. Hence, we are forced to investigate the evidence in this order: reliability, accuracy, clinical meaning.

Attention to data that are not proved to be relevant can introduce noise. In the worst case this noise confuses the clinical decision-making process. For example, a child is diagnosed with a kidney tumour. Chest CT shows three lung nodules, all less than 5 mm in diameter, and the nodules are not seen on the plain radiograph. The important question is this: Does the child have metastatic disease? In the absence of imaging criteria for lung nodules, the answer may be left to the treatment team’s discretion. Is it perceivable that the team’s decision is different if the primary tumour is very large and causes major anatomical distortion compared to if the tumour is small? Not impossible. But there is no evidence that the volume of the primary tumour at diagnosis is related to the likelihood of metastatic disease, nor that it is an independent prognostic factor. Hence, the attention to size represents noise that may bias the treatment decision.

This article is not a criticism of any particular individual or group. As part of an initiative to raise awareness of the importance of developing a strong evidence base for paediatric radiology, the objectives are to question ingrained practices (here, measuring tumours as a spinal reflex) and to promote the role of a theoretical framework as a facilitator for rational empirically based improvements. This article does not comprehensively review the field of paediatric oncological radiology. Instead, it presents examples that illustrate some important points.

## Size of the primary tumour

Two tumour groups illustrate some problems concerning radiologic measurements of primary tumours.

The Euro-E.W.I.N.G. (European Ewing tumour working initiative of national groups) EE 99 protocol (most recent version, 14 Sept 2010, not in the public domain) uses tumour volume (CT or MRI, not further specified; three dimensions with a spherical or cylindrical formula, not further specified) for allocation of children to randomisation groups. Of interest here, a child with localised disease and tumour ≥200 ml may be randomised to receiving high-dose therapy (busulfan–melphalan). The rationale is most likely found in the second German sarcoma study (inclusion 1986–1991, 152 patient data sets analysed), which reported a relative risk of about 2 for relapse in children with primary tumour ≥200 ml [2]. Children with primary tumour ≥100 ml had received intensified treatment in this study. Other works, however, did not provide clear justification for a size criterion. For example, Paulussen et al. [3] did not find evidence that size (volume more or less than 100 ml) was an independent prognostic factor (event-free survival or relapse rate) in children with primary lung metastases. A larger study of patients diagnosed 1978–1993 did show a negative prognostic effect of tumour volume ≥100 ml in both localised and metastatic disease [4]. Yet, the magnitude of this effect was not reported, volume data were only available in 454/796 patients and tumour volumes had been estimated from radiographs or CT images. The volume data used to devise the stratification system therefore seem to be somewhat deficient in rigour. In this context it is also interesting to note that no radiologist is listed as contributor to the Euro-E.W.I.N.G. protocol.

The staging system for non-metastatic rhabdomyosarcoma as proposed by the EpSSG (European paediatric soft-tissue sarcoma group) RMS-2005 protocol (most recent version, 10 Jan 2013, not in the public domain) is complicated, with eight defined subgroups. Imaging has a key role in the stratifications. For example, tumour maximum diameter must be ≤5 cm (conforming to the cutoff between T1 and T2 in the tumor-nodes-metastases [TNM] system) to be in the low-risk group or in subgroup D of the standard-risk group. The same cutoff is used for stratifications in synovial sarcoma and adult-type sarcomas, but these will not be discussed here. One immediate question: what does the protocol mean by “maximum dimension”? It is not a protocol requirement that volumetric acquisitions be made and datasets reconstructed to seek the maximum diameter. Because only axial, coronal and sagittal measurements are required, then tumour orientation could, in effect, influence the staging, which intuitively seems inappropriate. A study of intermediate-risk patients with rhabdomyosarcoma suggested that volume estimates are better than diameters for risk stratification [5]. In particular, a cutoff at 20 ml optimised the stratification. This is the volume of a spherical tumour with diameter 3.4 cm, which is well below the cutoff diameter (5 cm) in the EpSSG 2005 protocol.

Observation of a change in size of tumour, for example over the initial courses of chemotherapy, may seem to be a biologically rational marker. Although re-staging on response in size is not formalised, one could speculate that treatment teams do consider this information informally. Intuitively one would think that tumour growth or shrinkage during chemotherapy would be a marker of response. However, exceptions have been documented, for example in nephroblastoma, where tumour growth may be associated with differentiation (intermediate risk by the International Society for Paediatric Oncology [SIOP] renal tumour study criteria) and shrinkage with residual blastemal predominance (high-risk by SIOP criteria) [6]. This illustrates that macroscopic behaviour is not necessarily associated with histopathological behaviour. Again, formal evidence is required, even for what seems obvious.

## Lymph nodes

The absence of regional lymph node metastases is one criterion discriminating standard risk from high risk in rhabdomyosarcoma. Biopsy is recommended if there is clinical/radiologic uncertainty. There is, however, no imaging recommendation, except a preference for MRI. This matters because some pulse sequences are relatively more sensitive for lymph node metastases, e.g., diffusion-weighted imaging in adult cancer [7]. CT-PET is optional only, but would perhaps be a good candidate in a disease with relatively high rates (at least 15%) of nodal involvement [8], the presence of which dramatically influences the child’s prognosis. The alternative is to let size criteria define which lymph nodes most likely contain tumour deposits. There are two obvious problems involved. First, there are no agreed upper limits for normal lymph node dimensions by age group and anatomical region. Second, early metastatic states are always missed by size criteria. The latter problem is elaborated in the discussion of lung nodules in nephroblastoma.

## Unreliable numbers

Reliability means consistently good quality and performance. In the field of radiology we may specify this as consistently good quality of image acquisitions, consistent image representation of identical structures or processes (regardless of site and time) and precise interpretation within and between readers. Surprisingly few published papers investigate the reliability of radiologic findings. One may speculate that this category of articles is perceived, by authors and editors alike, to carry little prestige. Satisfactory reliability guarantees that images are of adequate quality (e.g., that they sufficiently and consistently discriminate neoplastic and non-neoplastic tissue) and demonstrates that readers can be trained to repeatedly and concertedly report (closely) consistent findings from the same image set.

Intra- and inter-observer variabilities are perhaps the two most comprehensive parameters for reliability, albeit they do not necessarily measure differences among imaging devices. Several statistics have been used to assess such variability, but only variability of scalar measurements shall be considered here. It is all too easy to find examples of various correlation statistics being employed for this purpose. There are (at least) three problems with this: (1) when two measurements are performed of the same structure, these measurements are destined to correlate. Anything else would come as a huge surprise. (2) Correlation statistics tell us very little about the degree of discrepancy, which clinically is the most important piece of information. (3) Correlation statistics do not specify any bias, for example whether the volumes of large tumours are less precisely estimated compared to small ones. One method that does convey all this relevant information is the one proposed by Bland and Altman [9]. Here the difference between paired measurements is plotted against the average of the same pair. This produces a visually informative scatterplot. Additionally, intervals (e.g., the ±2 standard deviation interval) of the differences provide information about expected variability, the mean of the measurement errors show any systematic bias, and a least-squares line reveals the potential non-random influence of the magnitude of measurements on errors.

As expected, there are few reports on reliability of tumour measurements. Those in existence are mostly discouraging. For example, at CT primary renal tumours measured independently by two experienced radiologists had a variability of ±500 ml [6]. Although most of this was contributed by the largest tumours, there was considerable variability among medium-size tumours, e.g., around the cut-off size for high-risk Ewing sarcoma (200 ml). A study of lung metastases in adults demonstrated a ±20% difference in volume estimates resulting from two CT scans performed with the same machine on the same day [10].

Studies of reliability should be the spine of radiologic research. It seems obvious that reproducibly of results across imaging devises, readers and time is a fundamental prerequisite for any test that aspires to offer clinical efficacy. The radiologic society at large may perceive that certain observations are bound to be reliable. However, as we have seen, even the humble measurement of tumour diameter is not. It follows, therefore, that size measurements, among several other imaging attributes, may not be suitable as discriminators until image acquisition and interpretation have been standardised and this standardisation has been proved to promote sufficient reliability. It is paradoxical, therefore, that size is nonetheless used as an independent staging parameter, e.g., in sarcoma, as seen above. Several undesirable possibilities may be conceived. For example, a tumour very close to the cut-off size (e.g., 5 cm in rhabdomyosarcoma and 200 ml in Ewing sarcoma) may be deliberately measured generously if the child clinically is thought to be in a high-risk group, or more conservatively if the disease is thought to be low-risk. Under this scheme the measurement in question has succumbed as an independent characteristic and is no longer of value to the patient. Poor reliability invites such practice, because “well, this measurement is not very reproducible anyway.” As a further consequence, when computing its prognostic validity at study-level, the measurement is confounded by other risk factors and is hence impossible to appraise as an independent variable. This is a thought (but not impossible) scenario where a hypothetically useful observation is rendered useless by poor reproducibility.

The message is twofold. First, a radiologic test (observation, measurement) is not a candidate for further investigation until it is scientifically plausible that it is reliable. This requires standardisation across imaging devises and among readers. It also calls for dissemination of results, for which researchers and editors are equally responsible. It must be expected from radiology representatives on oncological study groups that they consider reliability a fundamental requirement before implementing imaging measurements as part of any staging system. Absolute cut-off points require particularly high precision. Second, if imaging findings are to be part of a staging system then image acquisition and interpretation need to be clearly defined. Quality control needs to be implemented. This may be in the form of central review that (1) may help align the quality of imaging among participating centres and (2) may decrease the variability in reported quantities.

## What makes a lung nodule a metastasis?

Increasing sensitivity coupled with stationary specificity is recognised as a problem in the diagnosis of lung metastases [11]. In particular, false-positive results may cause upstaging. If benign (e.g., inflammatory) lung nodules are frequent in children with neoplastic disease, then this may be a real problem. Silva et al. [12] found lung nodules in 111/488 (23%) children with neoplastic disease at CT at diagnosis. There was probably little selection bias in this study, so the proportion seems high. Further, out of 26 biopsied nodules, 17 (65%) were benign. It therefore seems that a nodule, per se, is not necessarily a metastatic deposit, even in a child with cancer. Size criteria for discrimination of metastases and benign nodules have been proposed, so a discussion of lung nodules is apt in our context.

Lung metastases in rhabdomyosarcoma are defined as either at least one lung nodule ≥10 mm, or two or more *well*-*defined* nodules 5–10 mm, or 5 or more well-defined nodules <5 mm. The European Ewing protocol defines metastatic lung disease as at least one pulmonary/pleural nodule >10 mm, or more than one nodule >5 mm. Accurate diagnosis of metastatic disease is important because it may warrant high-dose chemotherapy. Interestingly, Paulussen et al. [3] found that in Ewing sarcoma the number of pulmonary metastases was not a prognostic factor whereas children with unilateral lung metastases were more likely to survive. Might this surprising finding have arisen from suboptimal diagnosis of lung metastases, i.e. false interpretation of nodules as metastases? This provocative interpretation may be supported by the finding that patients with metastases to lungs only do better than those with bone or bone marrow metastases [13]. It may be apposite to ask: what makes a nodule a metastasis?

In the SIOP 2001 renal tumour study, about 4% of all children with nephroblastoma had lung nodules seen on CT but not on chest radiograph. About two-thirds of these were treated as if they had metastatic disease. But compared with the one-third treated less intensively, there was no difference in survival [14]. One may interpret this as suggesting that small nodules are unlikely to represent metastases, hence that nodule size is a candidate criterion for discriminating benign and malignant lung nodules. Alternatively, small lung metastasis may resolve with non-metastatic treatment. There are several problems with this interpretation, however. First, chest CT was optional under the SIOP 2001 protocol, so the children who did have chest CT might represent a select group. Perhaps they were more likely to have some other risk factors? If so, the presence of lung nodules, whether malignant or benign, might have been less important overall. Second, the children with CT-only lung nodules were not randomised to receiving treatment for localised or metastatic disease. Hence, factors other than the CT findings most likely confounded the results. Finally, the CT-only criterion is a poor substitute for actual measurements. It must be less reliable by the fact that it is more difficult radiographically to detect a paraspinal nodule, say, than a nodule of the same size in the lateral mid-zone.

A similarly constructed study based on the National Wilms Tumor studies 4 and 5 demonstrated improved 5-year event-free survival among children with CT-only lung lesions who received additional chemotherapy [15]. However, the overall survival among these children was, as in the SIOP study, no different from survival among those treated for a local stage. The slight difference between these two studies probably only illustrates how difficult it is to gain accurate knowledge retrospectively from studies not originally incorporating good imaging questions. Too much bias is introduced by discretionary CT-scanning (selection bias), lack of clear staging criteria (information bias, because other data were allowed to influence care decisions) and non-randomisation (treatment bias).

We have seen that large clinical studies do not support a size criterion for lung nodules in nephroblastoma. Further, relatively large imaging studies specifically designed to investigate discrimination of benign and malignant lung nodules did not find nodule size to be of significant value [12, 16]. On the contrary, at primary staging in children with extra-pulmonary malignant disease, Silva et al. [12] found higher proportions of benign lesions both among nodules of diameter 5 mm or less and among nodules of diameter 10 mm and more. Hence, the available literature seems to contradict the notion that size (or other popular features like number, shape and border, for that matter) is a useful discriminator.

Theoretically, nodule size may be a candidate discriminator only if metastatic nodules are small (i.e. less than a set criterion) for a relatively short period of their lifetime. But because tumour growth is exponential, this seems unlikely. For example, let a spherical lung metastasis have a doubling time of 20 days, which seems reasonable [17]. When its diameter is 1 mm the volume is about 0.52 mm^3^, and at 10 mm the volume is about 520 mm^3^. Hence the volume has increased 1,000-fold. With the assumed doubling time, this would take about 200 days (20 × log 1,000/log 2). For comparison, an increase from 10 mm (4,200 mm^3^) to 20 mm (33,500 mm^3^; 8-fold increase in volume) would only take about 60 days (20 × log 8/log 2). From these results we see that if there are lung nodules, then there is a non-negligible probability that they will be small at diagnosis. This may be contrary to common belief and certainly challenges the approach of several protocol guidelines. There is, however, one potential theoretical justification for the current approach. If benign lung nodules at the time of staging are even more likely to be small, then a high-pass threshold on size would improve our specificity, albeit at the cost of lower sensitivity. Unfortunately, knowledge about this is very difficult to obtain because biopsy proof is not routinely obtained. Not knowing the likelihood of distribution by size for benign versus malignant nodules makes it impossible to construct a receiver operating characteristics curve and hence impossible to obtain knowledge about the predictive values of observed nodules based on size. The size criteria given in several treatment protocols, therefore, seem arbitrary at best and illogical in the worst case. It may be argued that the burden of lung nodules in itself may have predictive validity; however, as discussed above, outcome results from clinical trials are too confounded to allow proofs for this.

## Seeking a fitting face of validity

In comparing a test result (e.g., a measurement) with a reference standard, we assess the test’s validity. Validity, in the widest sense, can be defined as the quality of being *logically or factually sound*. For example, let us define an attribute: image-based estimates of tumour size. It may perhaps be proved plausible that the attribute is indeed very close to the actual measured sizes of excised lesions. If so, our chosen attribute has the quality of being factually sound. By definition it logically follows that it is valid.

However, this broad definition of validity does not consider clinical utility and may therefore lead us off-target. Our primary task is not to predict which size of specimen-pot needs to be available in the operating theatre. Rather, we aim at guiding the clinical care for the child. In oncological care this means to help in assigning (and, if needed, re-assigning) the patient to an optimal combination and sequence of therapeutic agents and modalities. Such staging (and re-staging), as performed in accordance with a set of rules, often contains imaging criteria, as illustrated above. What about the validity of the staging formula? Is it factually sound? Although it was straightforward to assess the *face validity* of a size estimate, we are now in deep water. We seek a trait against which we can evaluate the performance of staging, but what could this be? Certainly not tumour size, dissemination, genetic makeup, etc., because these attributes are themselves criteria that define the staging formula. In other words, the staging system is a construct of (all) currently available pieces of information. Hence it cannot be validated by assessing its strength of association with concurrent observables (*concurrent validity*). Similarly, the staging system can hardly be validated prospectively (*predictive validity*) because stage itself is fundamental to treatment allocation. Staging systems evolve by cumulatively incorporating qualities and quantities that are thought to represent (independent) prognostic factors. It is the breadth and comprehensiveness of these data, and the logical plausibility of the staging formula, that justify staging systems and may give them *content validity*.

It follows that using an established staging system as a reference standard for a (new) radiologic test is problematic in several ways. (1) The staging system is only a construct — it is not an observable of nature. Using it as a reference standard, we can only assess our test’s *construct validity*. A close association between the test and this reference standard, therefore, does not bring us closer to understanding a biological process — it only tells us whether we have a test that emulates a human-made scoring system. There is a benefit only if the new test is more reproducible or has lower costs (in the wider sense: cheaper, earlier, less-invasive) compared to the reference. (2) Few people set out to find a test that is not associated with a reference standard. Perhaps non-correlation is perceived as scientific failure? However, if we seek attributes with potential to add new dimensions to existing staging (or other scoring) systems, then non-association is exactly what we should go looking for. For example, amplification of the *MYCN* gene in neuroblastic tumours is not associated with the clinico–radiologic disease stage. If it were, it would have been history. But because it does not co-vary with stage, still being a predictive factor, it is an appealing quality and indeed useful as an additional dimension for stratifying treatment of neuroblastoma. As another example, one may find that change in standard uptake values at PET-CT is closely associated with histopathological response scoring in osteosarcoma. But so long as we continue to perform histopathological specimen examination (we will), this result in itself is of no particular benefit to the patient.

These few paragraphs illustrate that a reference standard and formal validity testing, per se, do not guarantee an effective measurement. The hope lies in its degree of dissociation from existing constructs and in its predictive power.

## A step in the right direction

The International Neuroblastoma Risk Group Staging System [18, 19] with related imaging guidelines [20] exemplifies a rational, size-ignorant stratification strategy. Rather than absolute size and extent, the local-regional staging is based on anatomical relations that chiefly define the likelihood (at initial diagnosis) that the primary tumour can be completely excised, a principle also used for staging of hepatoblastoma [21]. To concentrate the radiologic workup on the local behaviour of tumour appears to be rational because non-metastatic and metastatic disease are more accurately separated by other modalities (e.g., metaiodobenzylguanidine scintigraphy, histopathological examination of bone marrow samples). The more limited role for radiology is complemented by independent well-defined and proven risk factors (age, *MYCN* oncogene amplitude, chromosome 11q status, DNA ploidy, tumour histology and differentiation). The result is a multi-dimensional staging system that, at least theoretically, is preferable. The main weaknesses are perhaps that the image-defined risk factors are only based on consensus and that central review of imaging is not in operation.

## Summary of findings

This informal investigation has highlighted a few problems.

1. Oncological treatment protocols call for radiologic assessment of tumour size but may nevertheless fail to specify image acquisition and measurement techniques.
2. The perfect measurement does not exist, so reporting measurements without error estimates is meaningless. Yet we know very little about how measurements vary depending on tumour shape and location, imaging device and reader. If oncological staging systems use absolute cut-offs for size, then the magnitude of measurement variability around a cut-off point will determine the proportion of patients who are (falsely) assigned a higher or lower stage purely because of (random) measurement error.
3. Although tumour burden is ultimately linked to the mortality of neoplastic disease, we have little knowledge about the prognostic value of tumour volume at the time of diagnosis.
4. If clinical prognosis is a function of tumour size, then it must be assumed to be a continuous function. But size-dependent staging systems treat size as if it were discrete. Because size is not accurately and precisely estimated by imaging, staging of tumours close to the cut-off points is less predictable. Also, if measurements are consequently biased by other factors, including clinical, this may confound the interpretation of imaging data from clinical trials.
5. The size of lung nodules is used in staging systems, implying that size is a discriminator between benign and malignant lesions. Still, lack of knowledge about the sensitivity and specificity precludes any prediction of the clinical value of such assessments.

## The way forward

Revision of staging criteria may be difficult because oncological trials are pragmatic and cumulative. They are pragmatic because the staging systems are devised to have predictive (and not necessarily concurrent pathological) validity. For example, it may not be required that a lung nodule actually represents a metastasis if, for the cohort of patients, lung nodules are associated with poorer prognosis. Oncological trials are cumulative because new prognostic factors are added without the replacement of existing ones. Like with a house of cards, replacing a card in the middle is difficult. Reshuffling an entire floor is impossible. Nevertheless, oncological care is moving towards ever more refined stratification to improve survival and minimise adverse effects of treatments, and imaging will no doubt be a partner in this project. One fundamental requirement for a successful partnership is active contributions from radiologists during the development of treatment protocols. These inputs fall into two main categories.

First, there is a duty to explore new imaging qualities or quantities that may have prognostic value. If these turn out to mimic the existing staging system, they are of little value to development. The focus needs to be on validating prognostic factors that are independent of existing ones. Similarly, re-validation of existing measurements (e.g., primary tumour, lung nodules) is required to make staging more rational. As seen in neuroblastoma, accurate diagnosis of metastatic disease is crucial to more rational local staging. The addition of multimodal imaging, including nuclear medicine, is a promising route.

Second, but just as important, there is an urgent need for standardisation of imaging acquisition and interpretation. Measurement errors must be estimated and acknowledged, and absolute cut-off points (whether for size or other scalars) should only be used in conjunction with other criteria (e.g., metabolic imaging) unless the quantity in question can be obtained with sufficient precision.

## References

[1] Eisenhauer EA, Therasse P, Bogaerts J (2009). New response evaluation criteria in solid tumours: revised RECIST guideline (version 1.1). Eur J Cancer.

[2] Ahrens S, Hoffmann C, Jabar S (1999). Evaluation of prognostic factors in a tumor volume-adapted treatment strategy for localized Ewing sarcoma of bone: the CESS 86 experience. Cooperative Ewing Sarcoma Study. Med Pediatr Oncol.

[3] Paulussen M, Ahrens S, Craft AW (1998). Ewing’s tumors with primary lung metastases: survival analysis of 114 (European intergroup) cooperative Ewing’s sarcoma studies patients. J Clin Oncol.

[4] Cotterill SJ, Ahrens S, Paulussen M (2000). Prognostic factors in Ewing’s tumor of bone: analysis of 975 patients from the European intergroup cooperative Ewing’s sarcoma study group. J Clin Oncol.

[5] Rodeberg DA, Stoner JA, Garcia-Henriquez N (2011). Tumor volume and patient weight as predictors of outcome in children with intermediate risk rhabdomyosarcoma: a report from the Children’s Oncology Group. Cancer.

[6] Olsen ØE, Jeanes AC, Sebire NJ (2004). Changes in computed tomography features following preoperative chemotherapy for nephroblastoma: relation to histopathological classification. Eur Radiol.

[7] Fornasa F, Nesoti MV, Bovo C (2012). Diffusion-weighted magnetic resonance imaging in the characterization of axillary lymph nodes in patients with breast cancer. J Magn Reson Imaging.

[8] Crist WM, Anderson JR, Meza JL (2001). Intergroup rhabdomyosarcoma study-IV: results for patients with nonmetastatic disease. J Clin Oncol.

[9] Bland JM, Altman DG (1995). Comparing methods of measurement: why plotting difference against standard method is misleading. Lancet.

[10] Wormanns D, Kohl G, Klotz E (2004). Volumetric measurements of pulmonary nodules at multi-row detector CT: in vivo reproducibility. Eur Radiol.

[11] Weiser DA, Kaste SC, Siegel MJ et al (2013) Imaging in childhood cancer: a Society for Pediatric Radiology and Children’s Oncology Group joint task force report. Pediatr Blood Cancer 60:1253–1260 10.1002/pbc.24533 PMC4636336 23572212

[12] Silva CT, Amaral JG, Moineddin R (2010). CT characteristics of lung nodules present at diagnosis of extrapulmonary malignancy in children. AJR Am J Roentgenol.

[13] Kushner BH, Meyers PA, Gerald WL (1995). Very-high-dose short-term chemotherapy for poor-risk peripheral primitive neuroectodermal tumors, including Ewing’s sarcoma, in children and young adults. J Clin Oncol.

[14] Smets AM, van Tinteren H, Bergeron C (2012). The contribution of chest CT-scan at diagnosis in children with unilateral Wilms’ tumour. Results of the SIOP 2001 study. Eur J Cancer.

[15] Grundy PE, Green DM, Dirks AC et al (2012) Clinical significance of pulmonary nodules detected by CT and not CXR in patients treated for favorable histology Wilms tumor on national Wilms tumor studies 4 and 5: a report from the Children’s Oncology Group. Pediatr Blood Cancer 59:631–635 10.1002/pbc.24123 PMC3397278 22422736

[16] McCarville MB, Lederman HM, Santana VM (2006). Distinguishing benign from malignant pulmonary nodules with helical chest CT in children with malignant solid tumors. Radiology.

[17] Shackney SE, McCormack GW, Cuchural GJ (1978). Growth rate patterns of solid tumors and their relation to responsiveness to therapy: an analytical review. Ann Intern Med.

[18] Monclair T, Brodeur GM, Ambros PF (2009). The International Neuroblastoma Risk Group (INRG) staging system: an INRG task force report. J Clin Oncol.

[19] Cohn SL, Pearson AD, London WB (2009). The International Neuroblastoma Risk Group (INRG) classification system: an INRG task force report. J Clin Oncol.

[20] Brisse HJ, McCarville MB, Granata C (2011). Guidelines for imaging and staging of neuroblastic tumors: consensus report from the International Neuroblastoma Risk Group project. Radiology.

[21] Roebuck DJ, Aronson D, Clapuyt P (2007). 2005 PRETEXT: a revised staging system for primary malignant liver tumours of childhood developed by the SIOPEL group. Pediatr Radiol.

